# Daphnoretin relieves IL-1β-mediated chondrocytes apoptosis via repressing endoplasmic reticulum stress and NLRP3 inflammasome

**DOI:** 10.1186/s13018-022-03316-w

**Published:** 2022-11-16

**Authors:** Jianbo Zhou, Qinzhi Wang

**Affiliations:** 1 Department of Orthopedics, Xingshan People’s Hospital, Yichang, 443000 Hubei China; 2 Department of Orthopaedics, Huangshi Central Hospital, Affiliated Hospital of Hubei Polytechnic University, Edong Healthcare Group, No. 141, Tianjin Road, Huangshi, 435000 Hubei China

**Keywords:** Daphnoretin, Endoplasmic reticulum stress, Osteoarthritis, NLRP3

## Abstract

**Background:**

Osteoarthritis (OA), mainly caused by severe joint degeneration, is often accompanied by joint pain and dysfunction syndrome. Inflammatory mediators and apoptosis play key roles in the evolution of OA. It is reported that daphnoretin has significant antiviral and anti-tumor values. The present study aims at investigating the role of daphnoretin in OA.

**Methods:**

The OA mouse model was constructed by performing the destabilization of the medial meniscus through surgery, and the OA cell model was induced in ATDC5 chondrocytes with IL-1β (10 ng/mL) in vitro. Chondrocyte viability and apoptosis were measured by 3-(4,5)-dimethylthiahiazo (-z-y1)-3,5-di-phenytetrazoliumromide (MTT), Caspase-3 activity, and flow cytometry. The levels of COX-2, iNOS, TNF-α, IL-6, Bax, Bcl2, cleaved-Caspase3, endoplasmic reticulum stress (ERS) proteins (GRP78, CHOP, ATF6, and Caspase-12), and NLRP3-ASC-Caspase1 inflammasome were determined by quantitative real-time PCR or western blot. The concentrations of TNF-α, IL-6, and PGE2 were tested by enzyme-linked immunosorbent assay. The content of nitrates was detected by the Griess method. In vivo, morphologic differences in knee joint sections and the thickness of the subchondral bone density plate in mice were observed by hematoxylin–eosin (H&E) staining and safranin O-fast green staining.

**Results:**

Daphnoretin effectively choked IL-1β-induced chondrocyte apoptosis and facilitated cell viability. Daphnoretin dose-dependently abated ERS, inflammatory mediators, and the activation of NLRP3 inflammasomes in IL-1β-induced chondrocytes. What’s more, *in vivo* experiments confirmed that daphnoretin alleviated OA progression in a murine OA model by mitigating inflammation and ERS.

**Conclusion:**

Daphnoretin alleviated IL-1β-induced chondrocyte apoptosis by hindering ERS and NLRP3 inflammasome.

**Graphical abstract:**

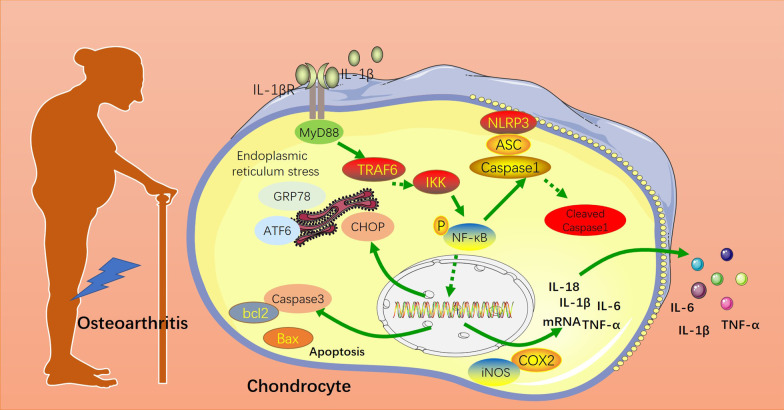

## Introduction

Osteoarthritis (OA) is a chronic and progressive joint disease accompanied by the reduction of chondrocytes and disintegration of the cartilage matrix. As a complicated degenerative disease affecting the whole joint, OA is pathologically associated with degeneration of knee cartilage, subchondral bone and synovium, meniscal damage, inflammation and fibrosis of the infrapatellar fat pad, which lead to joint pain and motor dysfunction [1, 2]. Although an increasing number of scholars are devoted to studying the molecular mechanism of OA, no breakthrough has been made due to its complex pathogenesis [3]. In a recent study, OA is reported to cause joint pain and deformity and contribute to cardiovascular diseases [4, 5]. Hence, it is crucial to probe the pathogenesis of OA and prevent its development.

Daphnoretin, with the molecular formula C_19_H_12_O_7_, is widely distributed in plant species, such as *Leguminosae* and *Glycosmis pentaphylla* (Rutaceae). As a natural dicoumarin component, daphnoretin has tumor-suppressive and antiviral effects [6]. For example, daphnoretin represses colon cancer cell migration and invasion by choking the Akt pathway [7]. Also, daphnoretin has a significant effect on cervical cancer [8]. Moreover, daphnoretin, one of the main components identified in *E. papyrifera* extracts, relieves bone loss in animal models of ovariectomy-induced osteoporosis [9]. However, little is known about the role of daphnoretin in OA.

The endoplasmic reticulum (ER) is the main place for cells to process proteins and store calcium ions, and it is susceptible to stress. Misfolding and unfolding protein aggregation in the lumen and disturbance of calcium balance are called endoplasmic reticulum stress (ERS) [10]. Multiple studies have testified that pranoprofen dampens ERS-mediated chondrocyte apoptosis to alleviate OA [11]. In addition, Chen D et al. found that salidroside, a promising therapeutic drug for OA, ameliorates OA by mitigating inflammation and ERS [12]. These findings suggest that weakening ERS and chondrocyte apoptosis can delay OA progression. However, it is not clear whether daphnoretin exerts protective effects against OA by modulating ERS.

The NLRP3 inflammasome is a high-molecular-weight protein complex containing NOD-like receptor protein 3 (NLRP3), the apoptosis-associated speck-like protein containing CARD (ASC) and Caspase-1. The NLRP3 inflammasome contributes to multiple processes, such as cell immune damage, apoptosis, and inflammation [11, 13]. Several studies have demonstrated that the NLRP3 inflammasome is extensively involved in OA development. For example, icariin mitigates OA by hindering the NLRP3/Caspase-1 signaling-mediated apoptosis [14]. Also, Wang et al. discovered that ursolic acid eases inflammatory reactions by inactivating the NF-κB/NLRP3 inflammasome, thereby preventing cartilage degeneration in OA [15].

Presently, we aimed to investigate the function and specific mechanism of daphnoretin in OA by establishing in vitro and in vivo OA models. Our data exhibited that daphnoretin suppresses IL-1β-induced chondrocyte apoptosis in vitro and improved cartilage injury of knee joint of the OA mouse model. Moreover, daphnoretin mitigated ERS and NLRP3 inflammasome activation both in vitro and in vivo. All over, we hope this study brings novel insights into OA treatment.

## Materials and methods

### Chondrocyte culture

ATDC5 chondrocytes were ordered from the Cell Center of the Chinese Academy of Sciences (Shanghai, China) and cultured in an RPMI1640 medium comprising 10% fetal bovine serum (FBS) and 1% penicillin/streptomycin (Invitrogen, CA, USA) at 37 °C with 5% CO_2_. The medium and FBS were provided by Thermo Fisher Scientific (MA, USA). During the cells’ logarithmic growth phase, 0.25% trypsin (Thermo Fisher HyClone, Utah, USA) was utilized for trypsinization and sub-culture.

### Drug treatment

Daphnoretin (Article No.: HY-N0699), the NLRP3 inflammasome inhibitor CY-09 (Article No.: HY-103666), and the ERS inhibitor 4-PBA (Article No.: Hy-a0281) were acquired from MedChemExpress (SH, USA). Daphnoretin, CY-09, and 4-PBA were dissolved in 10% DMSO. Then, they were diluted in 0.9% normal saline (NS). In vitro, chondrocytes were treated with IL-1β (PeproTech Inc., NJ, USA) alone (10 ng/ml) or in combination with different doses of daphnoretin (0, 0.5, 1, 2, 4, 8, 16, 32 µM) for 48 h, and/or received 1 nM of 4-PBA or CY-09 treatment in the medium.

### 3-(4,5)-Dimethylthiahiazo (-z-y1)-3,5-di-phenytetrazoliumromide (MTT) assay

ATDC5 chondrocytes at the logarithmic growth stage were seeded into 96-well plates at 5 × 10^4^ cells/mL and cultured for 24 h after trypsinization. Subsequently, the primary medium was discarded, and daphnoretin at different doses (0, 0.5, 1, 2, 4, 8, 16, 32, 64, 128, 256, and 512 µM) was added for further culture for 48 h. Five repetitive wells were set for each experimental group. Next, the medicated medium was discarded, and the MTT solution (Sigma-Aldrich, St. Louis, MO) (5 mg/mL) was added to each well and maintained for another four hours. Then, the supernatant was discarded, and 200 μL DMSO was added to each well for shaking incubation for 1.5 h. The absorbance was measured at 570 nm using a Thermo Multiskan FC microplate reader.

### Flow cytometry

ATDC5 chondrocytes in the logarithmic phase were collected to make single-cell suspensions (1 × 10^6^ cells/ml) and inoculated in a 25 cm^2^ culture bottle (each bottle contained 5 mL cell suspension). After cell adherence, the original medium was discarded. The experimental group was supplemented with the medium containing 0.3% FBS, and an equal volume of PBS was spiked into the control group. Then, the two mediums were incubated at 37℃ with 5% CO_2_ for 24 h. Afterward, the supernatant was harvested and cleared with cold PBS three times. Next, the cells were trypsinized with EDTA-free trypsin and collected. The remaining steps were performed following the Annexin V-PI Apoptosis Kit (Southern Biotechnology, Birmingham, Al, USA) instructions, and cell apoptosis was calculated by flow cytometry within 1 h.

### Quantitative real-time PCR (qRT-PCR)

Total RNA in the cells or tissues was isolated with the TRIzol reagent. Genomic DNA was removed using Ambion™ DNase I(RNase-free) (Cat.No.AM2222, Invitrogen). Then, 1 μg of the extracted total RNA was reversely transcribed into cDNA with the PrimeScript™ RT Reagent Kit (Invitrogen, Shanghai, China). The Bio-Rad CFX96 quantitative PCR system and SYBR Green qPCR Master Mix were purchased from MedChemExpress (Cat.No. HY-K0501, NJ, USA) that were employed for qRT-PCR, which was performed with predenaturation for 5 min at 95 °C, denaturation for 15 s at 95 °C, and annealing for 30 s at 60 °C. *Gapdh* was the internal reference of *Cox-2*, *iNOS*, *Tnf-α*, and *Il-6*, with the 2^(−ΔΔCt)^ method for statistics. qRT-PCR was implemented three times. The primers were synthesized by Guangzhou Ribo Biotechnology Co., Ltd. The primer sequences of each gene are exhibited in Table1.

**Table 1:** Primers used in this study

Genes	Primer sequences (5′ → 3′)
*Tnf-α*	Forward: AGCCCCCAGTCTGTATCCTT
	Reverse: CTCCCTTTGCAGAACTCAGG
*Il-6*	Forward: AGTTGCCTTCTTGGGACTGA
	Reverse: TCCACGATTTCCCAGAGAAC
*Cox2*	Forward: GCGAGCTAAGAGCTTCAGGA
	Reverse: TCATACATTCCCCACGGTTT
*inos*	Forward: CACCTTGGAGTTCACCCAGT
	Reverse: ACCACTCGTACTTGGGATGC
*Ga*pdh	Forward: AACTTTGGCATTGTGGAAGG
	Reverse: ACACATTGGGGGTAGGAACA

### Western blot (WB)

ATDC5 cells were washed with cold PBS three times. Then, 100~200 μL RIPA lysate (P0013K, Beyotime Biotechnology, Shanghai, China) was added to the cells for ultrasonic lysis in ice water, which was supplemented with protease inhibitor phenylmethanesulfonyl fluoride (PMSF, 1 mM) (Roche, Catalog No.10837091001). The protein content was determined by the Bradford method. 30 µg of total protein from each set of samples was separated by electrophoresis on 10% SDS-PAGE and then transferred to the polyvinylidene fluoride (PVDF) membranes (Millipore, Bedford, MA, USA). The membranes were blocked at 4 °C for one hour and then maintained at 4 °C overnight with the following primary antibodies (concentration 1: 1000): Anti-COX-2 antibody (ab15191), Anti-iNOS antibody (ab15323), Anti-Bax antibody (ab32503), Anti-cleaved caspase3 antibody (ab2302), Anti-Bcl2 antibody (ab182858), Anti-NLRP3 antibody (ab214185), Anti-ASC antibody (ab180799), Anti-Caspase-1 antibody (ab74279), Anti-GRP78 antibody (ab21685), Anti-CHOP antibody (ab11419), Anti-ATF6 antibody (ab122897), Anti-Caspase-12 antibody (ab62484), and Anti-GAPDH antibody (ab181602). After being rinsed with TBST twice, the membranes were kept at room temperature for one hour with fluorescein-labeled Goat anti-Rabbit IgG (ab205718, 1:2500). The above antibodies were all provided by Abcam (Cambridge, UK). After three washes, the membranes were exposed to ECL chromogenic agents (Millipore, Bedford, MA, USA) and imaged with a membrane scanner.

### Enzyme-linked immunosorbent assay (ELISA)

Followed by IL-1β and or daphnoretin treatment, the supernatants were collected and centrifugated (1000 rpm for 10 min at 4 °C). Determination of inflammatory factors (including TNF-α, IL-6, and PGE2) in the supernatant was carried out following the instructions of the related detection kits, including TNF-α (Cat.No. H052-1), IL-6 (Cat.No. H007-1-1), and PGE2 (Cat.No. H099-1) (Nanjing Jiancheng Bioengineering Institute, China).

### Caspase-3 activity test

The Caspase-3 activity detection kit (Cat.No. C1116, Beyotime, Shanghai, China) was adopted to test the caspase-3 activity in chondrocytes. 40 µL detection buffer, 50 µL sample, and lysis buffer were successively added to the 96-well plates, followed by the addition of 10 µL Ac DEVD pNA (2 mM) for treatment. After incubation at 37 °C for 60–120 min, the absorbance of Caspase-3-catalyzed pNA in different samples was monitored at 450 nm with a Thermo microplate reader (Multiskan FC).

### Establishment of the mouse OA model

Fifty 8–10-week-old *C57BL/6* male wild-type mice were ordered from the Animal Experiment Center of Wuhan University (Wuhan, China). Mice were randomized into (1) the sham operation group (sham, *N* = 15), (2) the OA group (received intraperitoneal injections of DMSO, *N* = 15), (3) the daphnoretin group (received intraperitoneal injection of daphnoretin at 10 mg/kg/d, *N* = 15), and (4) the OA + Daphnoretin group (received intraperitoneal injection of daphnoretin following OA modeling at 10 mg/kg/d, *N* = 15). The mice were administered with daphnoretin by intraperitoneal injection (daily for the first week postsurgery, and 3 days once during the next 7 weeks) for 8 weeks following surgery. Eight weeks after the surgery, the mice were sacrificed, and their knee joints were taken for histopathological examination. The mice in sham group were sham-operated. Destabilization of the medial meniscus (DMM) was performed to induce the mouse OA model [5]. Briefly, the mice were anesthetized, and their right knee capsules were cut from the medial patellar tendon in the OA group and the OA + Daphnoretin group. Microsurgical scissors were utilized to cut open the medial meniscus ligament. In contrast, in the sham group, only the joint capsule was cut open, and the medial meniscus ligament was not damaged. Mice were executed 8 weeks after the operation, and their knees were harvested for histopathological analysis. All animal tests were implemented as per the guidelines granted by the Ethics Committee of Huangshi Central Hospital, Affiliated Hospital of Hubei Polytechnic University (Approve No. HSCH-2020022, 2020).

### Histopathological analysis

The mice’s knee joints were fixed in 4% paraformaldehyde for 48 h, decalcified in 10% EDTA solution for one month, dehydrated with gradient ethanol, paraffin-embedded, and cut into 6 μM-thick sections. Hematoxylin–eosin (H&E) staining and the Modified Safranine O-Fast Green FCF Cartilage Stain (S–O) Kit (Solarbio, Beijing, China) were employed in the sham group, the OA group, and the OA + Daphnoretin group. Histomorphological scores were applied to each group of mice, and morphological differences in the stained knee sections in each group were inspected by the blind microscope method. The articular cartilage damage was evaluated according to the International Society for the Study of Osteoarthritis (OARSI) scoring system [11]. According to the previous description [16], the thickness of the inferior medial plate of cartilage from the staining sections was measured utilizing the Axio Vision software. Cartilage assessments were carried out by experimenters who were unaware of the animal experiments. TRAP staining was applied to examine the number of mature osteoblasts. The sections of mouse knee joints were subjected to incubation for two hours with the working solution of the TRAP staining kit (G1050, Wuhan Servicebio Technology Co., Ltd.). The slides were laundered three times during distillation and then stained with hematoxylin for five minutes. Afterward, the sections underwent dehydration, clearing and sealing with neutral gum. Five fields of view were randomly chosen for each section, and the staining results were observed using an Olympus DP71 light microscope.

### The Griess method

ATDC5 chondrocytes (3 × 10^5^ cells/well) were inoculated in 6-well plates and cultured. 24 h later, the cells were exposed to daphnoretin with different doses (1, 2, 4 µM) and/or CY-09 (1 nM) for 24 h before the IL-1β (10 ng/mL) treatment. The cells were then cultured for another 24 h and measured with a spectrophotometer to obtain the optical density. The concentration of nitric oxide (NO) was examined using the Griess reagent [17].

### Data analysis

The SPSS software (Version 20.0, Chicago, IL, USA) was employed for data analysis. All data were expressed as mean ± standard deviation. Student's t test was used for analyzing the differences of quantitative variables between two groups. For multi-group data, one-way ANOVA analysis was performed, followed by Bonferroni’s post hoc analysis. *P* < 0.05 presented statistical significance.

## Results

### Cytotoxicity of daphnoretin on chondrocytes

**Fig. 1:**
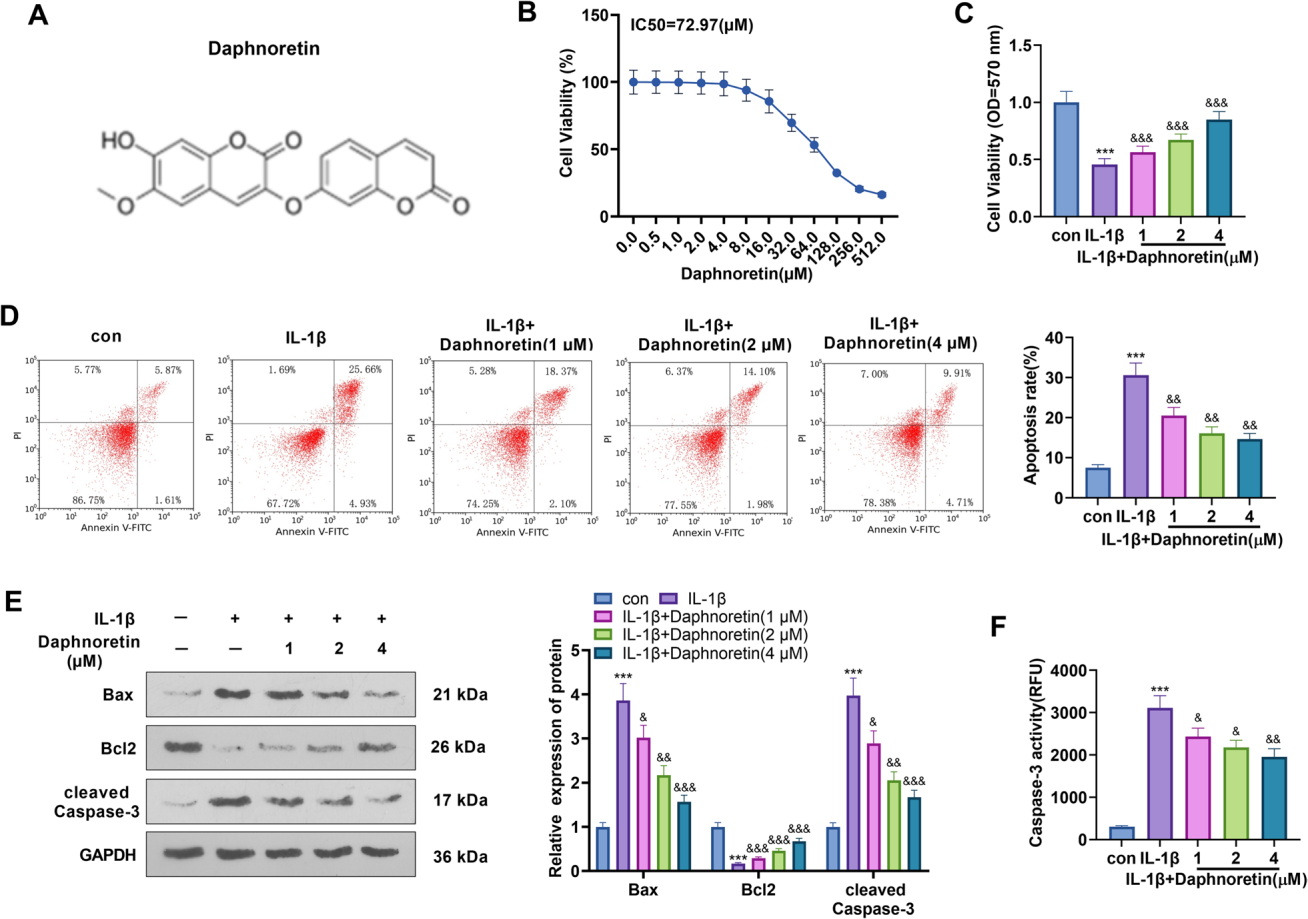
Effects of daphnoretin on chondrocyte toxicity in vitro. ATDC5 chondrocytes were treated with daphnoretin at different doses (0.5, 1, 2, 4, 8, 16, 32, 64, 128, 256, 512 µM) for 48 h. **A** Molecular structure of daphnoretin. **B** MTT assay was performed to check cell viability and determine the IC50 value of daphnoretin to be 72.97 µM. ATDC5 cells were treated with IL-1β (10 ng/mL) to induce the OA cell model, followed by treatment with daphnoretin (1, 2, 4 µM). **C** Cell viability was assessed by the MTT assay. **D** Flow cytometry was implemented to determine cell apoptosis. **E** Expression of Bax, Caspase-3 and Bcl2 was compared by WB. **F** The caspase-3 cell activity assay kit was adopted to examine the influence of daphnoretin on the apoptosis of mouse chondrocytes. *P < 0.05, **P < 0.01, ***P < 0.001 (vs. con group). &*P* < 0.05, &&*P* < 0.01, &&&*P* < 0.001 (vs. IL-1β group). *N* = 3

First, we treated ATDC5 cells with daphnoretin at different doses (0.5, 1, 2, 4, 8, 16, 32, 64, 128, 256, 512 µM) for 48 h to gauge the cytotoxicity of daphnoretin (the molecular chemical structure is displayed in Fig. 1A) on chondrocytes. Cell viability was tested by MTT, and an IC50 value of 72.97 µM in ATDC5 cells was determined for daphnoretin (Fig. 1B). As reported, IL-1β contributes to chondrocyte inflammation and apoptosis [18]. Therefore, IL-1β (10 ng/mL) was applied to construct the OA model in vitro*,* and ATDC5 cells were treated with daphnoretin (1, 2, 4 µM). Interestingly, After IL-1β treatment, cell viability was distinctly dampened, while the daphnoretin intervention increased cell viability dose-dependently versus the IL-1β group (*P* < 0.05, Fig. 1C). Flow cytometry results revealed that the chondrocyte apoptosis increased significantly after IL-1β stimulation, while it decreased dose-dependently after 48 h of pretreatment with daphnoretin (*P* < 0.05, Fig. 1D). WB outcomes displayed that the pro-apoptotic proteins Bax and cleaved-caspase3 were up-regulated, while the apoptosis-inhibitory protein Bcl2 was down-regulated after the IL-1β treatment. On the contrary, Bax and cleaved-caspase3 were down-regulated, while Bcl2 was up-regulated in the IL-1β + Daphnoretin group versus the IL-1β group (*P* < 0.05, Fig. 1E). The caspase-3 activity assay kit was applied to clarify the effects of daphnoretin on chondrocyte apoptosis. As exhibited in Fig. 1F, the Caspase-3 activity of chondrocytes was facilitated after IL-1β treatment, while it decreased notably after 48 h of daphnoretin pretreatment. Hence, daphnoretin enhanced the mouse chondrocyte viability and abated apoptosis.

### Daphnoretin abated IL-1β-induced ERS in chondrocytes

**Fig. 2:**
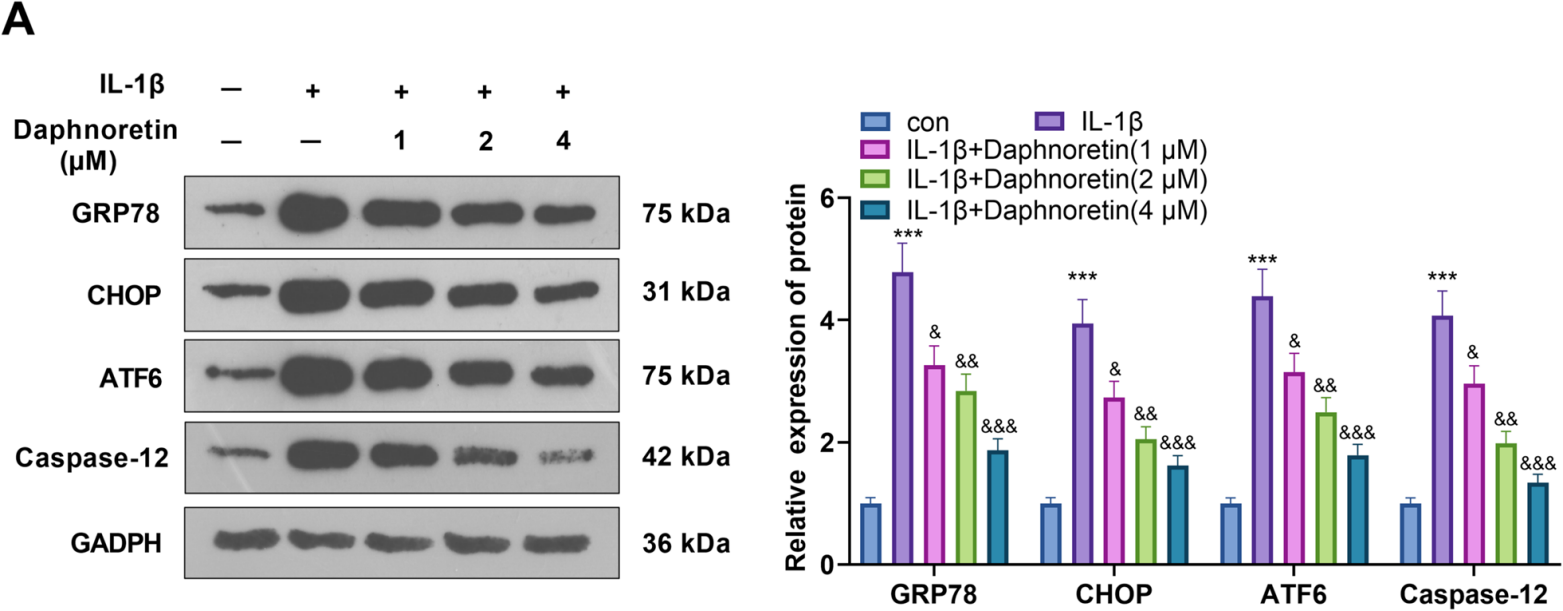
Daphnoretin abated the IL-1β-induced ERS in chondrocytes. IL-1β (10 ng/mL)-treated chondrocytes were treated with daphnoretin (1, 2, 4 µM). **A** The levels of GRP78, CHOP, ATF6, and Caspase-12 were detected by WB. ***P < 0.001 (vs. con group). &*P* < 0.05, &&*P* < 0.01, &&&*P* < 0.001 (vs. IL-1β group). *N* = 3

We treated IL-1β (10 ng/mL)-induced chondrocytes with daphnoretin at different doses (1, 2, 4 μM) to study the effect of daphnoretin on ERS in OA. The expression of ERS markers (GRP78, CHOP, ATF6, and Caspase-12) was compared by WB. The results illustrated that the levels of the above ERS markers were uplifted in the IL-1β group versus the control group, while daphnoretin dose-dependently attenuated their levels versus the IL-1β group (*P* < 0.05, Fig. 2A). These findings suggested that daphnoretin attenuated IL-1β-induced ERS in chondrocytes.

### Daphnoretin declined the expression of IL-1β-induced inflammatory mediators and the activation of NLRP3 inflammasomes in chondrocytes

**Fig. 3:**
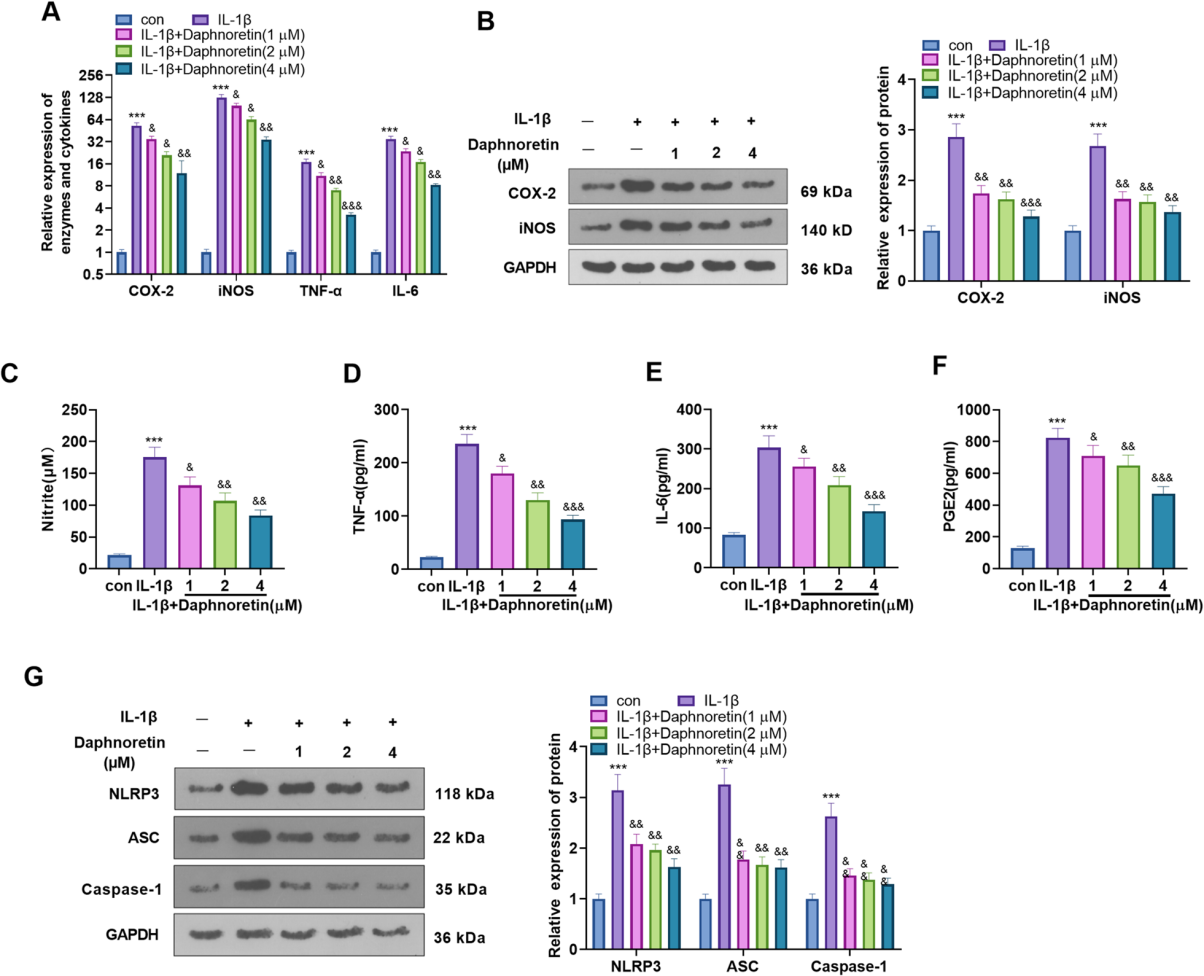
Daphnoretin repressed the expression of IL-1β-induced inflammatory mediators and activation of NLRP3 inflammasomes in chondrocytes. **A** qRT-PCR was applied to clarify the profiles of *Cox-2, inos, Tnf-α*, and *Il-*6. **B** WB was implemented to verify the profiles of COX-2 and iNOS. **C** The NO content was determined by the Griess method. **D**–**F** The concentrations of TNF-α, IL-6 and PGE2 in the chondrocyte supernatant were monitored by ELISA.** G** WB was applied to assess the NLRP3 inflammasome level. ***P < 0.001 (vs. con group). &*P* < 0.05, &&*P* < 0.01, &&&*P* < 0.001 (vs. IL-1β group). *N* = 3

Therefore, we conducted qRT-PCR to clarify the expression of *Cox-2*, *inos*, *Tnf-α*, and *Il-6*. It turned out that these inflammatory factors were up-regulated after IL-1β treatment, while they were attenuated after daphnoretin intervention (compared with the IL-1β group) (*P* < 0.05, Fig. 3A). WB was conducted to examine the levels of COX-2 and iNOS, and the results were consistent with those of qRT-PCR (*P* < 0.05, Fig. 3B). The outcomes of the Griess method demonstrated that the IL-1β treatment alone elevated the NO content. In contrast, compared with the IL-1β group, daphnoretin dose-dependently declined the NO content (*P* < 0.05, Fig. 3C). The concentrations of TNF-α, IL-6, and PGE2 in mouse chondrocyte culture supernatant were tested by ELISA. Interestingly, their concentrations were boosted by IL-1β treatment alone and reduced dose-dependently by daphnoretin compared to the IL-1β group (*P* < 0.05, Fig. 3D–F). Furthermore, the NLRP3 inflammasome level was determined by WB. The results displayed that IL-1β treatment alone down-regulated NLRP3, ASC, and Caspase-1, while daphnoretin intervention significantly diminished the effects of IL-1β (*P* < 0.05, Fig. 3G). These findings illustrated that daphnoretin abated IL-1β-induced inflammation and NLPRP3 activation in mouse chondrocytes.

### Daphnoretin alleviated IL-1β-treated chondrocyte apoptosis by repressing ERS

**Fig. 4:**
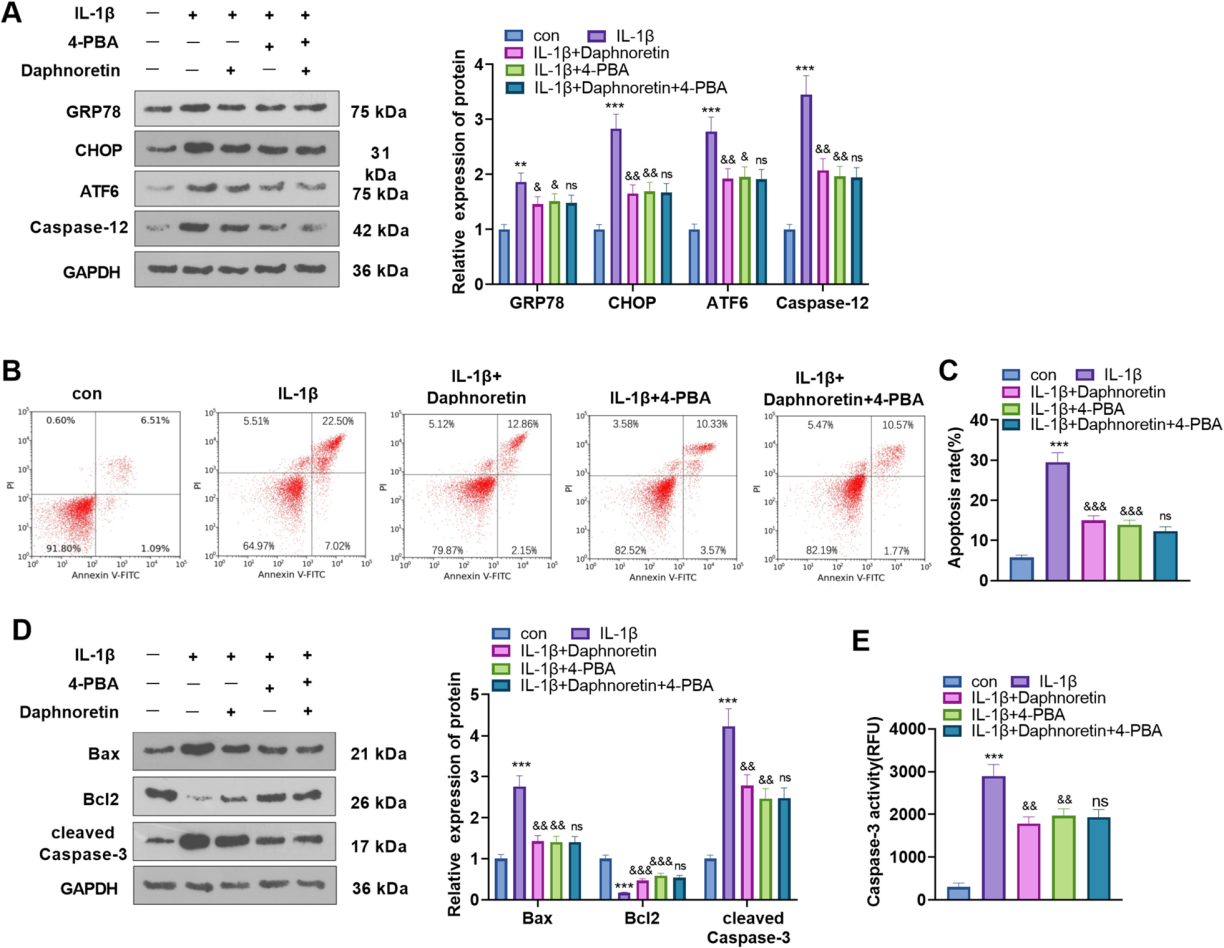
Daphnoretin alleviated IL-1β-mediated chondrocyte apoptosis by abating ERS. 10 ng/mL IL-1β was applied to mediate chondrocyte apoptosis, and then 4 μM daphnoretin and/or 1 nM 4-PBA were administered on this basis. **A** The expression of GRP78, CHOP, ATF6, and Caspase-12 was compared by WB. **B**, **C** Flow cytometry was implemented to determine apoptosis. **D** WB was conducted to verify the profiles of Bax, Caspase-3, and Bcl2. **E** The Caspase-3 cell activity detection kit was adopted to test the Caspase-3 activity. *P < 0.05, **P < 0.01, ***P < 0.001 (vs. con group). &*P* < 0.05, &&*P* < 0.01, &&&*P* < 0.001 (vs. IL-1β group). ns*P* > 0.05 (vs.IL-1β + 4-PBA group). *N* = 3

We administered 10 ng/mL IL-1β to induce apoptosis in chondrocytes, followed by 4 μM daphnoretin and/or 1 nM ERS inhibitor 4-PBA treatment to explore the specific mechanism by which daphnoretin suppressed chondrocyte apoptosis. WB was implemented to determine the expression of GRP78, CHOP, ATF6, and Caspase-12. As a result, compared with the IL-1β group, daphnoretin or 4-PBA application following IL-1β treatment down-regulated these ERS markers. Nevertheless, the expression of these ERS markers did not change after the combined intervention of daphnoretin and 4-PBA versus the IL-1β + 4-PBA group (*P* < 0.05, Fig. 4A). Flow cytometry manifested that daphnoretin or 4-PBA choked apoptosis, but there was no significant difference in apoptosis after the combined treatment of the two compared with that of the IL-1β + 4-PBA group (*P* < 0.05, Fig. 4B, C). Furthermore, WB outcomes uncovered that the treatment of daphnoretin or 4-PBA following IL-1β administration distinctly reduced the activities of Bax and cleaved caspase3 and up-regulated Bcl2 versus the IL-1β group. In comparison to the IL-1β + 4-PBA group, there was no significant difference in the expression of the apoptosis-related proteins after the combined intervention of the two (*P* < 0.05, Fig. 4D). The results of the caspase-3 activity assay also illustrated that the administration of daphnoretin or 4-PBA hindered the caspase-3 activity, while the two groups showed no significant difference after the combined utilization of daphnoretin and 4-PB versus the IL-1β + 4-PBA group (*P* < 0.05, Fig. 4E). Therefore, daphnoretin mainly inhibited chondrocyte apoptosis by abating ERS.

### Daphnoretin eased IL-1β-mediated chondrocyte inflammation by inactivating NLRP3 inflammasome

**Fig. 5:**
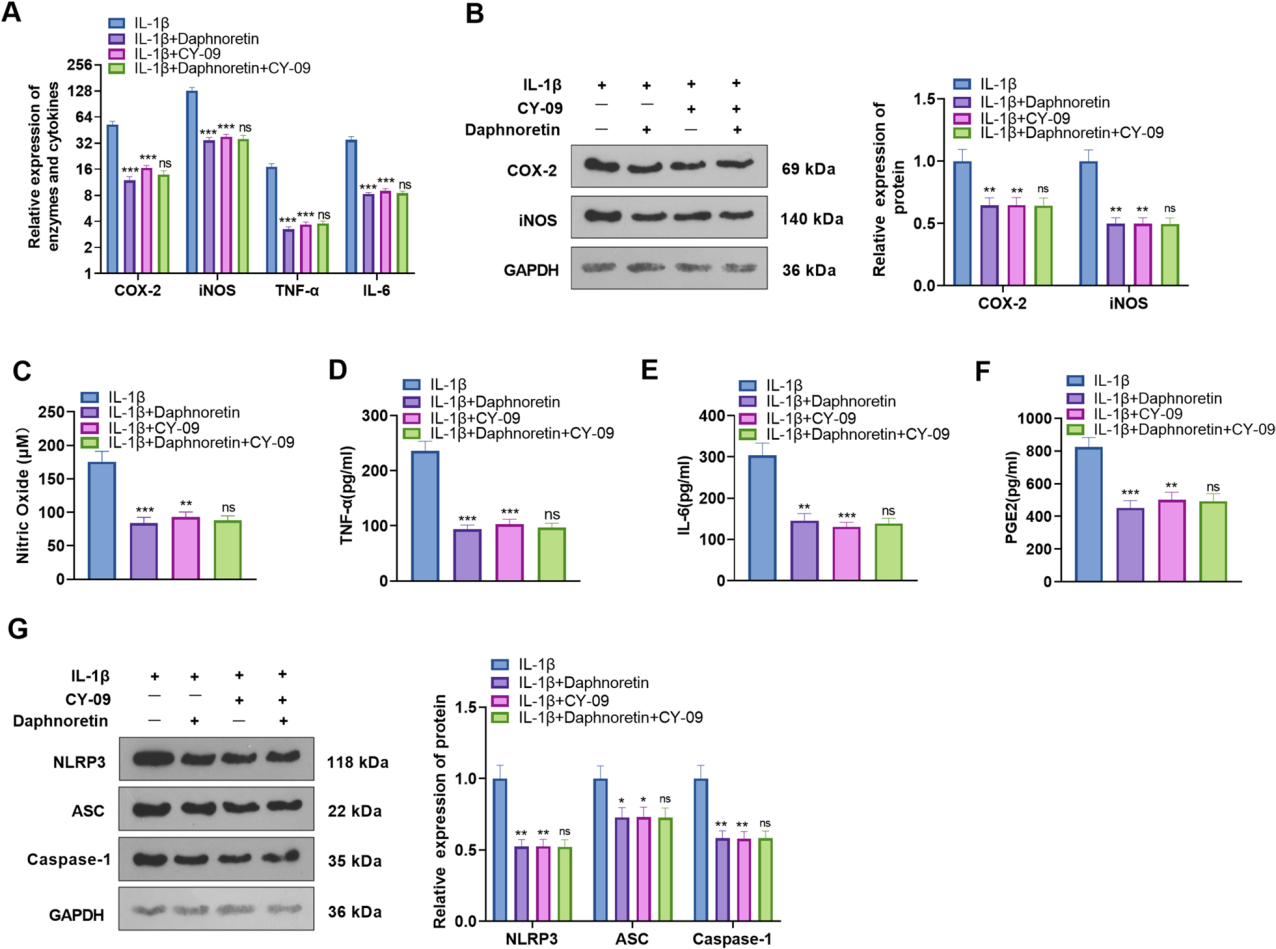
Daphnoretin eased IL-1β-mediated chondrocyte inflammation by inactivating NLRP3 inflammasomes. 10 ng/mL IL-1β was given to mediate chondrocyte apoptosis, and then, 4 μM daphnoretin and/or 1 nM CY-09 were administered on this basis. **A** qRT-PCR was implemented to clarify the expression of COX-2, iNOS, TNF-α, and IL-6. **B** The profiles of COX-2 and iNOS were monitored by WB. **C** The Griess method was applied to test the NO content. **D**–**F** The concentrations of TNF-α, IL-6, and PGE2 in the chondrocyte supernatant were examined by ELISA. **G** WB was performed to verify the NLRP3 inflammasome level. *P < 0.05, **P < 0.01, ***P < 0.001 (vs.IL-1β group). ns *P* > 0.05 (vs. IL-1β + CY-09 group). *N* = 3

Chondrocytes were treated with 10 ng/mL IL-1β, followed by administration of 4 μM daphnoretin and/or 1 nM NLRP3 inhibitor CY-09. qRT-PCR was employed to test the expression of COX-2, iNOS, TNF-α, and IL-6. It was discovered that both daphnoretin and CY-09 hindered the expression of these inflammatory mediators versus the IL-1β group. The combination of daphnoretin and CY-09 exerted no more inhibitory effects on those inflammatory mediators versus the IL-1β + CY-09 group (*P* < 0.05, Fig. 5A). Additionally, the protein levels of COX-2 and iNOS were monitored by WB. As a result, the expression of COX-2 and iNOS was attenuated by daphnoretin or CY-09, while there was no significant difference in their expression after the combined intervention of the two versus the IL-1β + CY-09 group (*P* < 0.05, Fig. 5B). The results of the Griess method displayed that compared with the IL-1β group, the application of daphnoretin or CY-09 resulted in a decrease in NO levels, while NO levels in the IL-1β + CY-09 group were not significantly distinct from those in the IL-1β + Daphnoretin + CY-09 group (*P* < 0.05, Fig. 5C). Furthermore, the expression of TNF-α, IL-6, and PGE2 in the chondrocyte supernatant as well as NLRP3 inflammasome was gauged. The results hinted that daphnoretin or CY-09 treatment markedly attenuated the expression of TNF-α, IL-6, PGE2 and the NLRP3-ASC-Caspase1 inflammasome versus the control group (Fig. 5D–G). However, there was no significant difference in the expression of pro-inflammatory cytokines and NLRP3 inflammasome in the IL-1β + Daphnoretin + CY-09 group as compared to the IL-1β + CY-09 group (*P* > 0.05, Fig. 5D–G). Therefore, daphnoretin is a potent inhibitor of NLRP3 inflammasome in IL-1β-mediated chondrocytes.

### Daphnoretin alleviated OA in mice

**Fig. 6:**
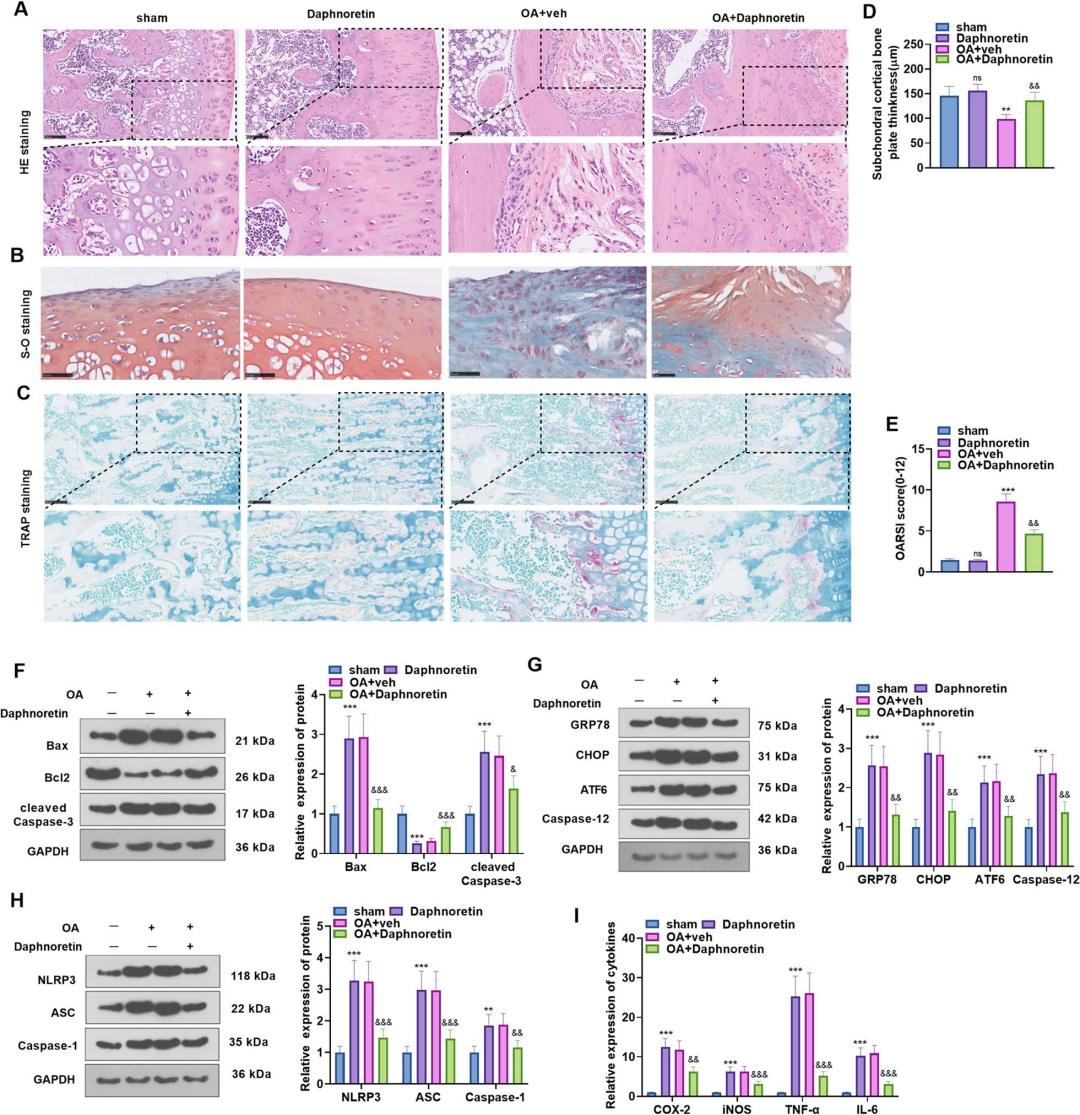
Daphnoretin alleviated OA in mice. The OA mice were intraperitoneally injected with 10 mg/kg of daphnoretin. **A**–**C** H&E staining, Safranine O-Fast Green staining and TRAP staining were adopted to observe the morphological differences in the knee sections and the thickness of the subchondral cortical bone plate in mice. **D** Quantitative analysis of the thickness of the subchondral cortical bone plate in mice. **E** Quantitative analysis of OARSI scores. **F** WB was adopted to evaluate the expression of Bax, Bcl2, and cleaved Caspase-3. **G**–**H** The profiles of GRP78, CHOP, ATF6, Caspase-12 and NLRP3 inflammasomes were compared by WB. **I**: qRT-PCR was performed to assess the expression of COX-2, iNOS, TNF-α, and IL-6 in the cartilage tissues. **P < 0.01, ***P < 0.001 (vs. sham group). &*P* < 0.05, &&*P* < 0.01, &&&*P* < 0.001 (vs. OA + veh group). *N* = 5

To test the function of daphnoretin in OA mouse models, we adopted H&E staining, safranin O-fast green staining and TRAP staining to observe the morphological differences in mouse knee joint sections. As a result, the articular cartilage surface was worn away, with severe cartilage erosion and reduced cartilage thickness in the OA group compared to the sham group. However, the daphnoretin (10 mg/kg) treatment repaired the articular cartilage surface and increased the thickness of cartilage and subchondral cortical bone plate in the OA mouse model (Fig. 6A, B). The number of TRAP-positive cells was increased in the OA group. After daphnoretin treatment, TRAP-positive cells were decreased compared with the OA group (Fig. 6C). Then, the thickness of the subchondral cortical bone plate and the OARSI score were calculated for evaluating the degree of joint damage. It turned out that the thickness of the cortical bone plate was reduced, whereas the OARSI score was significantly elevated in the OA group. In OA + Daphnoretin group, the thickness of the cortical bone plate and OARSI score were both significantly improved (compared with the OA group*, P* < 0.05, Fig. 6D–E). WB outcomes revealed that Bax and cleaved-caspase3 in the cartilage tissues were up-regulated, while Bcl2 was down-regulated in the OA + Daphnoretin group versus the sham group, whereas the results were mostly reversed in the OA + Daphnoretin group (*P* < 0.05, Fig. 6F). WB results also hinted that GRP78, CHOP, ATF6, Caspase-12 and NLRP3 inflammasome were up-regulated in the OA group versus the sham group. Nevertheless, the daphnoretin intervention reduced the above effects (*P* < 0.05, Fig. 6G–H). Furthermore, we performed qRT-PCR to monitor the expression of *Cox-2*, *inos*, *Tnf-α*, and *Il-6* in the cartilage tissues. We observed that the mRNA levels of *Cox-2*, *inos*, *Tnf-α*, and *Il-6* were all significantly up-regulated in the OA group, which were abated by daphnoretin (Fig. 6I). These conclusions suggested that daphnoretin improved OA by hindering ERS and NLRP3-inflammasome-mediated inflammation.

## Discussion

As a chronic painful joint disease, OA has seriously affected the patients' quality of life [19]. Currently, treatment for OA is largely focused on pain relief, with surgery being the mainstay of treatment for advanced OA. Although various non-surgical methods have been widely introduced, they can only partially alleviate the symptoms of OA with limited efficacy [20–22]. Hence, finding more effective drugs for OA treatment is urgent. Daphnoretin has been reported to have antiviral and anti-tumor activities [23]. Here, we testify that daphnoretin reduces inflammation and chondrocyte apoptosis by inactivating ERS and NLRP3 inflammasome, thereby preventing OA. This research provides an important reference for drug-targeted therapy of OA.

Natural compounds from traditional Chinese medicine are highly effective, with few side effects and low toxicity. Those characteristics make them effective in the treatment of OA. For example, resveratrol, a most well-known polyphenolic stilbenoid present in grapes, mulberries, peanuts, rhubarb, and other plants, inhibits OA disease progression by upregulating SIRT1 [24]. Curcumin is a natural compound of *Curcuma longa L.*, and it curbs the advancement of OA [25]. Daphnoretin has been found with several biological activities and is used in the treatment of arthritis, tuberculosis and tumors [26]. Our data supported that daphnoretin markedly attenuated IL-1β-induced apoptosis. Actually, enhanced chondrocyte apoptosis, which causes progressive destruction of articular cartilage, is an evident feature in the occurrence and prognosis of OA [27]. Followed by IL-1β stimulation, TRAF6 was upregulated and causes NF-κB signaling pathway activation. Increased NF-κB phosphorylation induces upregulated inflammatory mediators and mitochondrial dysfunction, thus leading to chondrocyte apoptosis [28, 29]. As reported, artesunate abates IL-1β-mediated inflammation and apoptosis through inactivation of the NF-κB signaling in chondrocytes and delays OA evolution in mice [30]. Fortunately, we observed potent antiapoptotic effect in chondrocytes followed by daphnoretin treatment. Moreover, daphnoretin abated the IL-1β-induced production of PGE2, NO, TNF-α and IL-6, which are all involved in chondrocyte apoptosis and cell viability decline [31–34]. *In vivo* experiments showed that daphnoretin alleviated the pathological injury of the knee joint in OA mice. These findings suggested that daphnoretin has protective effects in OA.

Chondrocytes are the primary cells of articular cartilage and are therefore the main target of inflammatory stimuli. The inflammation caused by OA induces apoptosis in chondrocytes, further aggravating OA [35]. Additionally, inflammation-induced ERS triggered chondrocyte apoptosis to expedite the development of OA, and the inhibition of ERS significantly alleviates OA [36, 37]. In the context of ERS induction, chondrocytes exhibit suppressed growth and increased apoptosis [38]. Notably, there is growing evidence that ERS is positively associated with chondrocyte apoptosis during OA [39]. Several ERS-sensing proteins, such as CHOP and GRP78, are crucial in chondrocyte apoptosis and cartilage degeneration [40, 41]. Excessive ERS has been reported to up-regulate the expression of CHOP and GRP78 and activate the Caspase pathway, which is mainly in charge of apoptosis [42]. Besides, AFT6, a membrane-bound transcription factor, is activated when unfolded proteins accumulate in the endoplasmic reticulum, and it further activates ERS genes (e.g. GRP78) [43]. Notably, PGE2 [44], TNF-α [45] and IL-6 [34] all play a role in activating ERS. Our results confirmed that daphnoretin restrains the expression of ERS markers dose-dependently. By reducing the ERS with 4-PBA, we discovered that the expression of ERS markers and apoptosis-related proteins did not exhibit significant differences after daphnoretin intervention on this basis. Thus, daphnoretin serves as a potential ERS inhibitor in attenuating chondrocyte apoptosis.

In recent years, the NLRP3 inflammasome has gradually become a research hotspot. Several studies have linked abnormal activation of NLRP3 inflammasomes to chronic aseptic inflammation [46, 47]. NLRP3 inflammasomes can be activated by lipopolysaccharide (LPS) stimulation. Following LPS or IL-1β treatment, TLR4/NF-κB pathway is activated and then promotes the formation of NLRP3-ASC-Caspase1 inflammasome complex [48, 49]. Activated inflammasome can activate and cleave Caspase-1, thereby inducing the maturation and secretion of IL-1β and IL-18, which in turn boost the transcriptional activity of NF-κB and interferon regulatory factors (IRFs) and contribute to the inflammatory response [14]. On the other hand, the NLRP3 inflammasome complex induces apoptosis by cleaving the GSDMD to form GSDMD-N [50]. As reported, inhibiting NLRP3 inflammasome-mediated inflammation and apoptosis alleviates OA [51]. Here, we testified that daphnoretin dose-dependently lowered the contents of COX-2, iNOS, TNF-α, and IL-6 and inactivated the NLRP3 inflammasome. CY-09 was further adopted to choke the NLRP3 expression, and it was found that there was no significant difference in the expression of the aforementioned inflammatory mediators and NLRP3 inflammasomes following daphnoretin application on this basis. These findings indicate that daphnoretin represses OA by inactivating NLRP3 inflammasome.

**Fig. 7:**
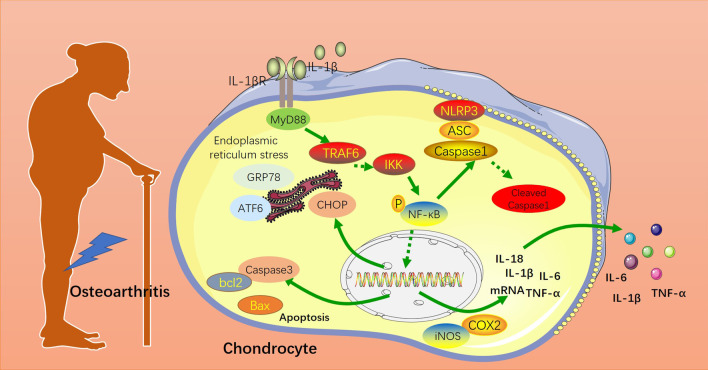
Schematic illustration of daphnoretin in OA by mediating endoplasmic reticulum stress and NLRP3 inflammasome

To sum up, this study confirms that daphnoretin promotes chondrocyte viability, reduces apoptosis, and protects bones by inactivating ERS and NLRP3 inflammasome, which provides a more powerful basis for OA treatment (Fig. 7). However, further studies should be performed for validating the mechanism of ERS and NLRP3 inflammasome in daphnoretin-mediated protection in vivo.

## Data Availability

The data sets used and analyzed during the current study are available from the corresponding author on reasonable request.
